# Improving the Measurement of Disease Activity for Patients with Rheumatoid Arthritis: Validation of an Electronic Version of the Routine Assessment of Patient Index Data 3

**DOI:** 10.1155/2015/834070

**Published:** 2015-11-08

**Authors:** Ruthie M. Chua, John N. Mecchella, Alicia J. Zbehlik

**Affiliations:** ^1^Dartmouth-Hitchcock Medical Center, Lebanon, NH, USA; ^2^Dartmouth-Hitchcock Medical Center, Geisel School of Medicine at Dartmouth, Hanover, NH, USA; ^3^Dartmouth-Hitchcock Medical Center, The Dartmouth Institute for Health Policy & Clinical Practice, Geisel School of Medicine, Hanover, NH, USA

## Abstract

*Introduction*. An electronic Routine Assessment of Patient Index Data 3 (RAPID 3) was incorporated into our electronic health records (EHRs) which did not replicate the visual presentation of the paper version. This study validated the electronic RAPID 3 compared to the paper version. *Methods*. Rheumatoid arthritis (RA) patients (*n* = 50) completed both the electronic RAPID 3 online in the week prior to and a paper version on the day of their clinic visit. *Results*. Paired *t*-test showed no significant difference (*p* value = 0.46) between versions. *Conclusion*. The electronic version of RAPID 3 is valid and can be easily integrated in care of RA patients.

## 1. Background

Patient-reported outcome measures (PROMs) are common tools used to inform about and improve patient care. They provide valuable longitudinal information about a patient's experience with his or her health and treatment. Because the patient is the only source of information, it is not subjected to interobserver variability and may be more sensitive for monitoring disease activity over time. PROMs also engage patients to become more active participants in monitoring their health [1–3].

As electronic health records (EHRs) proliferate, there is increased interest in electronic versions of PROMs that can be easily deployed through the web or touch screen tablets and seamlessly integrated into the patient chart. Automated PROMs increase compliance by decreasing administrative burden and facilitate efficient collection and interpretation of data. However, many of the more commonly used tools that have been validated as paper tools have not been tested in their electronic versions to see if they produce different results. Consequently, the migration of PROMs from the paper to the electronic version requires evidence of measure equivalence especially if there is modification of the visual presentation such as splitting a questionnaire into multiple screens or using scrollbars [4, 5].

The routine assessment of patient index (RAPID 3) is a validated PROM for rheumatoid arthritis (RA) that evaluates a patient's physical function, pain, and global health estimate. Previous studies that compared RAPID 3 to Clinical Disease Activity Index (CDAI) and Disease Activity 28 (DAS 28) showed significant correlation in disease activity severity. RAPID 3 is scored 0–30 with scores >12 indicating high disease activity, 6.1–12 moderate disease activity, 3.1–6 low disease activity, and ≤3 disease remission [6–8].

Dartmouth-Hitchcock Medical Center is currently using Epic version 2014 (Epic Systems Corporation, Verona, WI) as its EHR platform. An electronic version of the RAPID 3 was developed that could be integrated into routine patient care and research. Due to technical constraints when the questions were incorporated into the EHR, there were several significant formatting modifications which changed the layout of the questionnaire. The aim of this study was to validate the performance characteristics of the EHR version of RAPID 3 compared to the paper version in adult RA patients at an academic medical center.

## 2. Method

**Figure 1:**
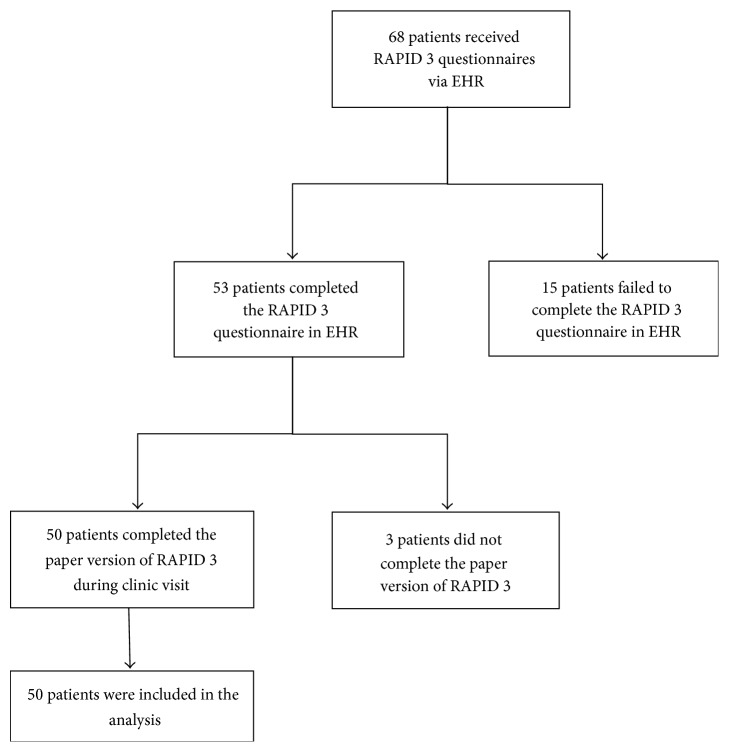
Flowchart of patient recruitment.

Our study was granted exemption by Committee for the Protection of Human Subjects of Dartmouth College. Between March and June 2014, we prospectively identified patients ≥ 18 years old with seropositive and seronegative rheumatoid arthritis who were presenting to the rheumatology clinic at Dartmouth-Hitchcock Medical Center. We verified the diagnosis via chart review and included only those who are active users of the patient portal of the EHRs and had at least 2 visits related to rheumatoid arthritis. The patient portal allows patients web-based access to their medical records, provides a confidential channel for physicians and patients to communicate, and allows providers to send online questionnaires for patients to complete prior to their appointment. Electronic versions of the RAPID 3 were sent to patients via the patient portal one week prior to their clinic appointment. They were alerted by an email reminder and a telephone call that a questionnaire was available to them. They completed the questionnaire online anytime in the week prior to the appointment. On the day of the clinic visit, we identified patients who had completed the electronic version and asked them to complete the paper version of the RAPID 3 in the reception area. We included the first fifty sequential patients to complete both the paper and electronic versions in the study (Figure 1). We excluded patients who did not complete the questionnaire for either the paper or electronic version. The EHR automatically calculated and interpreted the disease activity score for the electronic RAPID 3. Results of the paper form were manually calculated. A paired *t*-test was used to compare samples, with *p* values of ≤0.05 considered significant.

## 3. Results

Of the fifty patients included in the study, 66% were female. The average age was 58.8 years old (range 30–81 years) and average time lapse between answering the electronic and paper versions was 4.2 days (range 0–7 days). A paired *t*-test did not show statistically significant difference between the mean total RAPID 3 of the paper and electronic versions (*p* = 0.46). This was also true for each component score for RAPID 3. The means for the total RAPID 3 for both paper and EHR versions were 9.57 (±6.45) and 9.75 (±6.46), respectively, indicating moderate disease activity (Table 1).

## 4. Discussion

We found no significant difference in responses between the electronic and paper versions of the RAPID 3 questionnaire. The data entered by patients in electronic RAPID 3 is automatically stored, calculated, and interpreted in the EHR, thus eliminating the need for manual calculation, manual entry, and storage of paper scores. Additionally, patients may answer questions at home, and this can save time in a busy clinic and improve patient flow. Prior electronic scores can be easily reviewed and this facilitates comparison of disease activity over time. The EHR has the ability to prompt patients when questionnaires are incomplete and improved compliance has been previously demonstrated with electronic versions [9, 10]. It is interesting to note that the mean RAPID 3 showed moderate disease activity. However, some of the patients may have chronic pain and functional impairment which could drive their RAPID 3 score. Hence, the RAPID 3 is more useful for monitoring disease activity over time.

Our study has several limitations. While a randomized crossover study design for measuring equivalence was considered, due to the large catchment area and long travel distances for appointments, we felt that this was not feasible in our setting. In a randomized crossover study, participants are randomly assigned to either the paper or electronic version of the questionnaire. After a predetermined period of time, they are assigned to the other version and the results are then compared [4]. Although the sample size was relatively small, the study was adequately powered to show a difference in a paired *t*-test. There was no qualitative component to the study to help understand patients' personal experiences in terms of acceptance, convenience, and preference when answering the questionnaires. A review of our panel shows that only 52.5% of our 1130 RA patients have an active EHR patient portal. Therefore, our study population may not be representative of all RA patients in the practice as the sample only included participants active on the patient portal and, therefore, represent a computer-literate subpopulation of patients. This may introduce selection bias, as we do not know how patients who use the portal differ from other RA patients or why the 15 patients did not complete the electronic version. There was a mean of 4.2 days in between the electronic and paper versions. We were unable to complete a sensitivity analysis to see if longer times between completing the electronic and paper versions affected the scores.

Most rheumatologists still rely on nonquantitative assessments of RA activity despite studies showing that quantitative tools are associated with tighter control and thus better outcome [11–13]. In our academic center, documentation is mostly narrative, and if quantitative tools are used, they are not standardized in formatting and not easily searchable. By validating the electronic version, practitioners now have a convenient tool to monitor disease activity and ultimately improve quality of care. The electronic version of the RAPID 3 continues to be utilized in our clinic and we have expanded its use to include a tablet which can be used at point of care for those patients without EHR portal access. The information gathered from electronic RAPID 3 can be retrieved easily for individual patient care, clinical research, quality improvement, and innovations in care delivery.

## 5. Conclusion

Scores on an electronic version of RAPID 3 are not statistically significant from the validated paper version and can streamline patient reported data collection to improve the quality of patient care in a rheumatology clinic.

## Figures and Tables

**Table 1:** Comparison of paper and electronic health record versions of RAPID 3.

	Mean (±standard deviation)		Paired *t-*test *p* value
	Paper	EHR	
Physical function	1.87 (1.91)	1.85 (1.83)	0.85
Dress yourself	0.40 (0.64)	0.42 (0.67)	0.36
Get in and out of bed	0.46 (0.61)	0.34 (0.59)	0.41
Lift full cup or glass to mouth	0.20 (0.53)	0.18 (0.56)	0.37
Walking outdoors on flat ground	0.40 (0.67)	0.40 (0.67)	0.50
Wash and dry your entire body	0.42 (0.64)	0.48 (0.68)	0.36
Bend down to pick up clothing from floor	0.48 (0.68)	0.50 (0.68)	0.50
Turn regular faucet on and off	0.20 (0.53)	0.20 (0.53)	0.50
Get in and out of a car, bus, train, or airplane	0.54 (0.65)	0.50 (0.58)	0.23
Walk two miles or three kilometers, if you wish	1.20 (1.18)	1.20 (1.20)	0.46
Participate in recreational activities and sports	1.28 (1.03)	1.34 (1.08)	0.37

Pain score	3.86 (2.64)	4.03 (2.65)	0.27
Patient global assessment	3.84 (2.75)	3.87 (2.80)	0.65

Total	9.57 (6.45)	9.75 (6.46)	0.46
